# Effect of the salts of deep ocean water on the production of cordycepin and adenosine of *Cordyceps militaris*-fermented product

**DOI:** 10.1186/s13568-015-0140-5

**Published:** 2015-08-14

**Authors:** Yu-Ping Hung, Jyh-Jye Wang, Bai-Luh Wei, Chun-Lin Lee

**Affiliations:** Department of Life Science, National Taitung University, 369, Sec. 2, University Rd., Taitung, 950 Taiwan, ROC; Department of Nutrition and Health Science, Fooyin University, Kaohsiung, Taiwan, ROC

**Keywords:** *Cordyceps militaris*, Deep ocean water, Ions, Cordycepin, Adenosine

## Abstract

*Cordyceps militaris* is a type of entomogenous fungi and has been widely used as a medicinal fungus in Asia. Cordycepin produced by *C. militaris* has also been found to protect the liver. Moreover, deep ocean water (DOW) was proven to increase the functional compounds of functional fungi-fermented products. However, the regulation of the metals in DOW is still unclear. Therefore, this study investigated the effect of DOW and certain major ions on the production of cordycepin and adenosine of *C. militaris.* The results indicated that, compared with using ultra-pure water (UPW), using DOW to cultivate *C. militaris* in a submerged culture increases the production of biomass and adenosine (*p* < 0.05). In the results of solid culture, the concentration of DOW exhibits a dose effect on cordycepin production. DOW contains ions that can improve the effectiveness of cordycepin, such as Mg^2+^, Na^+^, Ca^2+^, Fe^2+^, and NO_3_^−^, whereas the ion Cl^−^ features an inhibitory effect. Moreover, Mg^2+^, Na^+^, K^+^, Ca^2+^, Fe^2+^, and SO_4_^2−^can increase the production of adenosine, whereas Cl^−^ cannot. However, the synthetic water made from various types of sodium salts (MgCl_2_, NaCl, KCl, CaCl_2_, FeCl_2_) had nearly the same effect on cordycepin production as that of DOW.

## Introduction

Deep ocean water (DOW) is characterized by low temperature, cleanness, and affluent production of inorganic nutrients and minerals. In recent years, because of its medicinal value, DOW has been extensively studied. Previous studies have noted that DOW has several health benefits, such as mitigating skin inflammation (Hataguchi et al. 2005), lowering blood lipids (Miyamura et al. 2004), reducing blood sugar (Hwang et al. 2009a), and reducing obesity (Hwang et al. 2009b). Moreover, DOW can be used to increase production of functional ingredients of microorganisms (Lee 2015). Studies have found that DOW can increase the functional ingredient production of monascin and ankaflavin of red mold dioscorea as well as triterpenoids, polysaccharide, and flavonoids of *Antrodia camphorata* (Lee et al. 2011; Wang et al. 2013a). Red mold dioscorea cultured by DOW lowers blood lipids and obesity more than that cultured by reverse osmosis water (ROW) and ultra-pure water (UPW) (Lee et al. 2011; Wang et al. 2013b). Moreover, compared with *A. camphorata* cultured by ROW, *A. camphorata* cultured by DOW protects the liver more effectively (Wang et al. 2013a). However, functional ions and the effects of ions in DOW on the growth and metabolites production of the functional fungi are still unclear incurrent. It should be an important topic for the application of DOW in future.

Recently, *Cordyceps militaris* has been regarded as an alternative to *Ophiocordyceps sinensis*. *C. militaris* has a similar functional composition to that of *O. sinensis* (Wang and Yao 2011). *C. militaris* also has antioxidant (Yu et al. 2006), antiinflammatory (Won and Park 2005), and antitumor effects (Yoo et al. 2004) as well as enhances sexual performance (Chang et al. 2008). According to relevant studies, the activity levels of the essential amino acids, cordycepin, *Cordyceps* polysaccharide, and superoxide dismutase of artificially cultured *C. militaris* are all higher than those of wild *O. sinensis* (Dong et al. 2012).

This study used *C. militaris* BCRC 32219 as the testing strain and focused on solid culture to examine how various culture mediums and concentrations of DOW affect the production of adenosine and cordycepin of *C. militaris.* This study also analyzed the effect of the major ions in DOW on the production of adenosine and cordycepin of *C. militaris* by adding only one single type of salt.

## Materials and methods

### Chemicals

Yeast mold (YM) agar and broth were purchased from Difco Laboratories Co. (Detroit, MI, USA). Magnesium, sodium, potassium, calcium, zinc and iron standard solutions were purchased from Showa Chemical Co. (Tokyo, Japan). Ethanol (95 %) was purchased from Taiwan Tobacco and Liquor Co. (Taipei, Taiwan). The other chemicals purchased from Sigma Chemical Co. (St. Louis, MO, USA).

### The preparation of DOW, SW, and various salts water

The concentrated DOW purchased from the Taiwan Yes Deep Ocean Water Co. (Hualien, Taiwan) was pumped from a depth of 670 m in the Pacific Ocean near the Eastern Taiwan and processed though the electrodeionization. According to our previous study, DOW including 20.65 mg/L Mg^2+^ was defined as onefold DSW (Wang et al. 2013a). In this study, 30-fold DOW (including 619.5 mg/L Mg^2+^) was prepared by the dilution of concentrated DOW (including 43,400 mg/L Mg^2+^) with UPW. The concentrations of the trace elements and minerals in 30X DOW included 619.5 mg/L Mg^2+^, 327 mg/L Na^+^, 132 mg/L K^+^, 5.13 mg/L Ca^2+^, 8.55 µg/L Fe^2+^, 1.156 mg/L nitrate, 649 mg/L sulfate, and 1.898 g/L chloride.

In the preparation of the various metals salts solution, Mg(NO_3_)_2_, NaCl, KCl, CaCO_3_, and (NH_4_)_2_Fe(SO_4_)_2_ solution were prepared according to the equal Mg^2+^, Na^+^, K^+^, Ca^2+^, Fe^2+^ concentrations in 10X-DOW, respectively. The five salts solutions were further mixed for the preparation of the synthetic water of mixed salts (Salt-SW). In the preparation of the sodium salts solution, NaNO_3_, Na_2_SO_4_, Na_3_PO_4_ and NaCl solutions were prepared according to equal SO_4_^2−^, PO_4_^3−^, NO_3_^−^, and Cl^−^ concentration in 30X or 60X DOW, respectively. These four sodium salts solutions were mixed for the preparation of a synthetic water of mixed sodium salts (Na-SW). In the preparation of the nitrate salts solution Mg(NO_3_)_2_, NaNO_3_, KNO_3_, Ca(NO_3_)_2_, Fe(NO_3_)_2_·6H_2_O solutions were prepared according to equal Mg^2+^, Na^+^, K^+^, Ca^2+^, and Fe^2+^ concentrations in 30X DOW, respectively. In the preparation of the chloride salts solutions, MgCl_2_, NaCl, KCl, CaCl_2_, FeCl_2_ solutions were prepared according to equal Mg^2+^, Na^+^, K^+^, Ca^2+^, and Fe^2+^ concentrations in 30X DOW, respectively. These five chloride salts solutions were further mixed for the preparation of a synthetic water of mixed chloride salts (Cl-SW).

### Microorganism and seed cultures

*C. militaris* BCRC 32219 was purchased from the Bioresource Collection and Research Center (Hsinchu, Taiwan). *C. militaris* was maintained on YM agar at 24 °C and transferred to fresh medium for 10 days intervals. Seed cultures were prepared by transferring a loopful of colony from YM agar slant into a 500-mL Hinton flask containing 100 mL medium (3 g/L yeast extract, 5 g/L malt extract, 10 g/L peptone, 3 g/L dextrose). The cultures were incubated at 28 °C and 100 rpm for 5 days. After that, inoculum sizes 5 % was transferred to submerged or solid cultured substrate.

### Submerged fermentation of *C. militaris* in DOW or UPW

Submerged fermentation was carried out using a 500-mL Hinton flask containing 100-mL medium (3 g/L yeast extract, 5 g/L malt extract, 10 g/L peptone, 3 g/L dextrose in UPW or DOW). The cultures were incubated at 28 °C for 5 days at 100 rpm. After submerged culture, mycelium and filtrate were separated using filter paper. The mycelium was dried by freeze dryer and then weighted. The dried mycelium powder and fresh filtrate was analyzed for intracellular and extracellular β-1,3 glucan, respectively.

### Solid fermentation of *C. militaris* in DOW or ROW

Thirty grams oat substrates was soaked in 30 mL UPW, DOW, SW, or various salts water, and then was autoclaved for 20 min at 121 °C in a 500-mL glass bottle. After being cooled, the substrate was inoculated with a 10 % (v/w) seed culture medium. The inoculated substrate was cultured at 24 °C for 20 days in a dark incubator. After dark culture, *C. militaris* was then cultured at 14 °C under a 12 h light:12 h dark cycle (light on at 6:00) for 60 days. After fermentation, the crushed and dried product was used for the experiments.

### Determination of β-1,3 glucan

The selective aniline blue reaction was employed to detect the existence of β-1,3 glucan. The method described by the previous studies (Wood and Fulcher 1984; Young and Jacobs 1998) were followed with some modifications. The sample was dissolve with 0.3 N NaOH and stirred at ambient temperature for completely dissolve. The pH of the sample solution was then adjusted to 11.5 ± 0.1 by adding 1 N HCl and the volume was made to 10 mL using Na_2_HPO_4_–NaOH buffer (pH 11.5 containing 0.5 M NaCl). A 0.2 mL of sample was reacted with 0.1 mL aniline blue (1 mg/mL) for 2 h at ambient temperature. The excitation and emission wavelength were set at 395 and 495 nm, respectively.

### Determination of cordycepin and adenosine

The powder of *C. militaris*-fermented product (0.1 g) was extracted respectively with 1 mL of methanol at 50 °C for 1 h. The extracts (10 %, w/v) were further filtered with 0.45 μm pore size filter and analyzed by HPLC (Model L-2130, Hitachi Co., Tokyo, Japan) on a C_18_ column (25 cm × 4.6 mm i.d., 5 μm, Luna^®^, Phenomenex, Torrance, CA, USA) using the gradient elution. HPLC was performed according to the method described previously (Yu et al. 2007) in triplicate. Cordycepin and adenosine were separated by gradient elution using the mobile phase with the composition of water–methanol (95.0/5.0 to 58.4/41.6 in 20 min, v/v). The flow rate was set at 0.8 mL/min. Cordycepin and adenosine were detected using a photodiode array detector (Model L-2455 DAD, Hitachi Co.) set at 260 nm and full wavelength.

### Statistical analysis

Data are expressed as mean ± standard deviation. Analysis of variance by Duncan’s test and Pearson’s product-moment correlation coefficient test were determined using SPSS version 10.0 software (SPSS Institute, Inc., Chicago, IL, USA). Differences with *p* < 0.05 were considered statistically significant.

## Results

### Effect of deep ocean water on the production of mycelium and certain functional ingredients under submerged culture

This study investigated the effect of DOW on the production of *C. militaris* mycelium and certain functional ingredients of *C. militaris* under submerged culture. As presented in Table 1, compared with ultra-pure water (UPW), DOW enhanced the production of *C. militaris* mycelium by 23 % (*p* < 0.05), which significantly increased the production of adenosine by 18 % (*p* < 0.05). However, DOW did not significantly affect the production of intracellular and extracellular β-1,3-glucan (*p* > 0.05). Moreover, under the submerged culture, the experimental results of this study revealed that no cordycepin was produced in the fermentative liquid and mycelium of UPW and DOW.

**Table 1:** Effect of DOW on the biomass, cordycepin, adenosine, intracellular β-1,3-glucan, and extracellular β-1,3-glucan production of *C. militaris* in submerged culture

	Biomass (g)	Cordycepin (mg/g)	Adenosine (mg/g)	Intracellular β-1,3-glucan (mg/g)	Extracellular β-1,3-glucan (mg/mL)
UPW	0.57 ± 0.07	–^a^	1.77 ± 0.16	13.35 ± 2.38	0.020 ± 0.004
DOW	0.70 ± 0.02*	–^a^	2.08 ± 0.09*	15.83 ± 2.06	0.021 ± 0.001

Data are presented as mean ± SD (n = 3).

* Indicated the significant difference (*p* < 0.05) as compared with UPW.

^a^Under detection limit.

### Effect of various deep ocean water concentrations on production of cordycepin and adenosine in *Cordyceps militaris*-fermented products

**Fig. 1:**
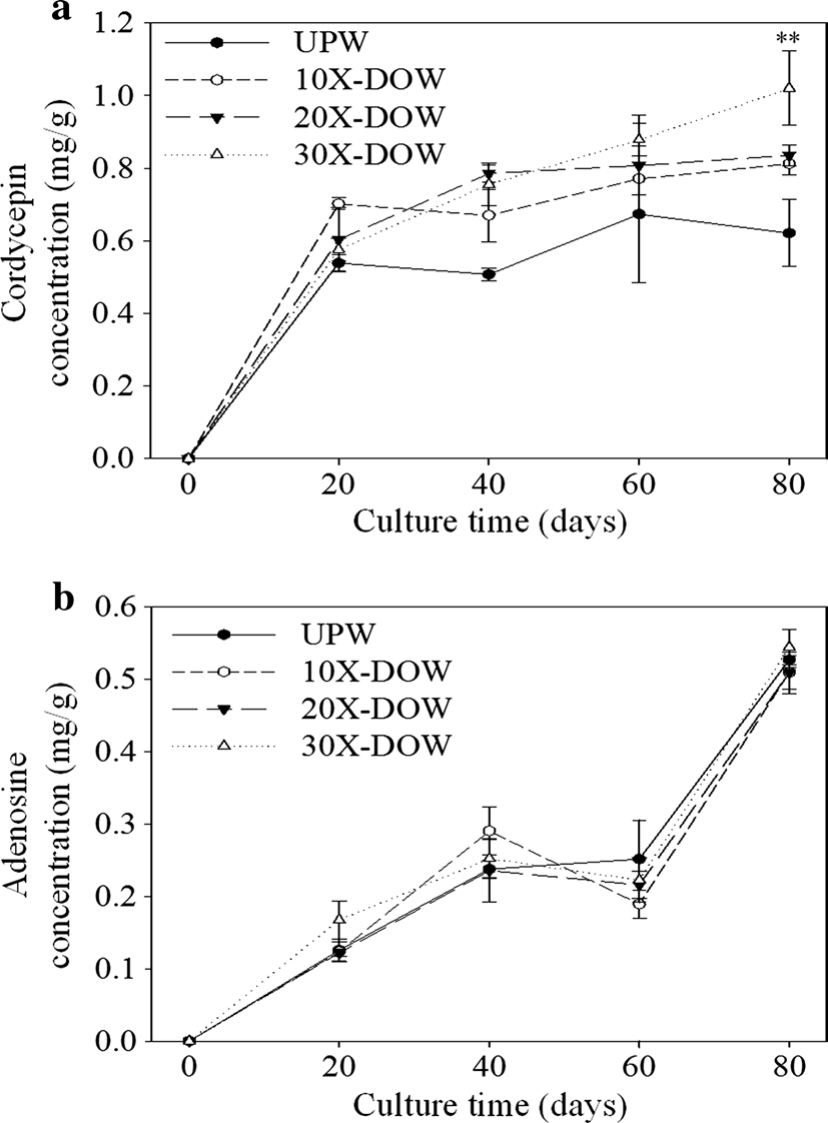
Effect of various concentration of DOW on the cordycepin (**a**) and adenosine (**b**) production of *C. militaris*-fermented oats in solid culture. Data are presented as mean ± SD (n = 3). ** indicated the significant difference (*p* < 0.01) as compared with UPW.

Cordycepin cannot be produced by cultivating *C. militaris* in submerged culture. Therefore, this study focused on solid culture and explored the effect of various factors on production of the functional ingredients of *C. militaris.* For this phase, the DOW of various concentrations was used as the water source for *C. militaris*-fermented production. The effect of DOW on the production of adenosine and cordycepin was also analyzed. Figure 1a shows that after 60 days’ fermentation, the *C. militaris*-fermented products with UPW, 10X-DOW, or 20X-DOW sources had increased cordycepin production; however, the increase was lower than that of the *C. militaris*-fermented products with the 30X-DOW source. When the fermentation was extended to 80 days, the *C. militaris*-fermented product with the 30X-DOW source had the highest cordycepin production. This cordycepin production was higher than that of the 20X-DOW, 10X-DOW, and UPW sources by 22 % (*p* < 0.01), 26 % (*p* < 0.01), and 64 % (*p* < 0.01), respectively, displaying a dose effect.

Figure 1b presents the production state of adenosine. When DOW of various concentrations was used as the water source for *C. militaris*-fermented production, the production of adenosine at various time points did not differ significantly (*p* > 0.05).

### Effect of the major metal ions of deep ocean water on production of cordycepin and adenosine of *Cordyceps militaris*-fermented products

**Fig. 2:**
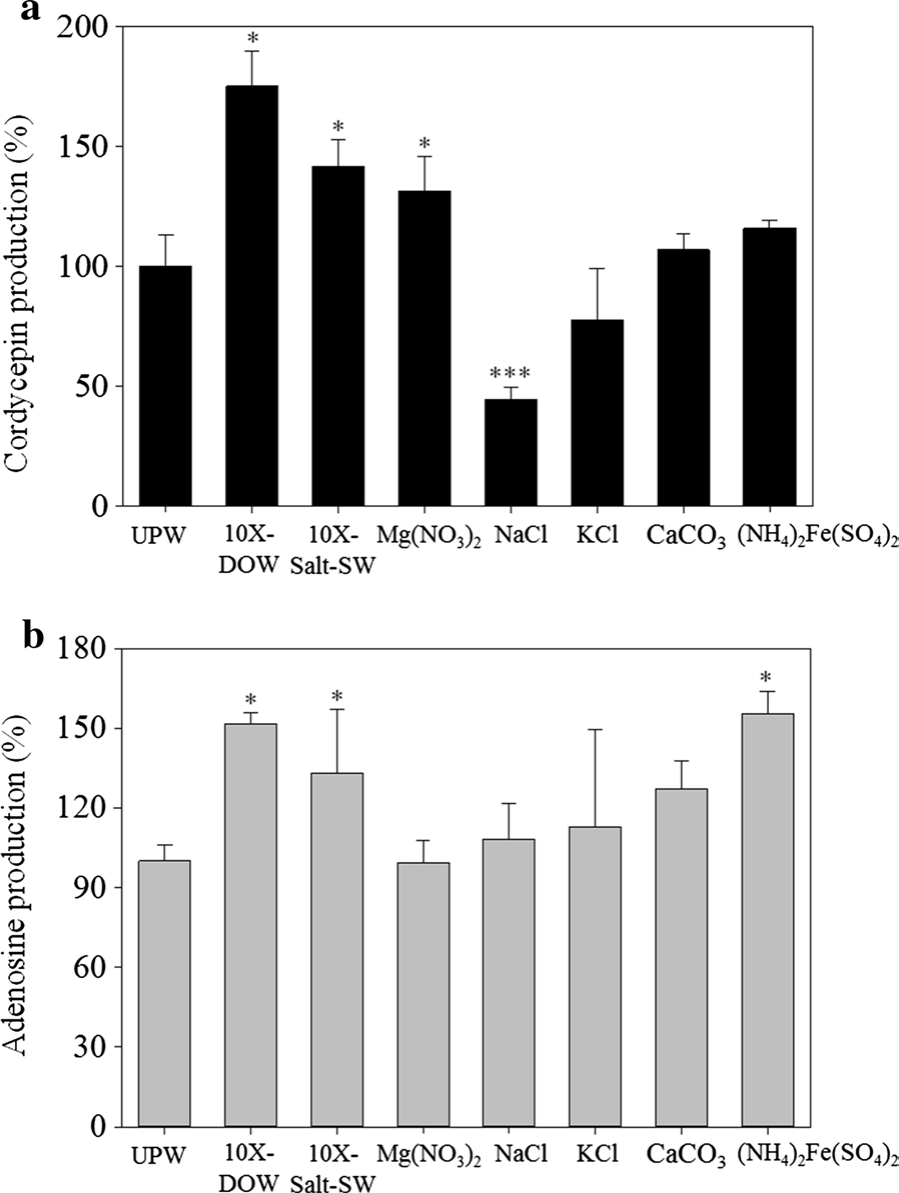
Effect of major metal ions in DOW on the cordycepin (**a**) and adenosine (**b**) production of *C. militaris*-fermented oats in solid culture. The concentration of Mg^2+^, Na^+^, K^+^, Ca^2+^, Fe^2+^ in Mg(NO_3_)_2_, NaCl, KCl, CaCO_3_, and (NH_4_)_2_Fe(SO_4_)_2_ were equal to that in 10X-DOW, respectively. Salt-SW was a mixture of the five salt solutions. Data are presented as mean ± SD (n = 3). *, *** indicated the significant difference (*p* < 0.05, *p* < 0.001, respectively) as compared with UPW.

DOW is rich in the concentration of Mg^2+^, Na^+^, K^+^, Ca^2+^, Zn^2+^, and Fe^2+^, which is possible to enhance the production of the functional metabolites. Therefore, the effect of the major metal ions in DOW on the production of cordycepin and adenosine were further analyzed in this study. Figure 2a shows the effect of DOW, Salt-SW, and various salts solution on cordycepin production. The concentration of Mg^2+^, Na^+^, K^+^, Ca^2+^, Fe^2+^ in Mg(NO_3_)_2_, NaCl, KCl, CaCO_3_, and (NH_4_)_2_Fe(SO_4_)_2_ were equal to that in 10X-DOW, respectively. The five salts solutions were mixed for the preparation of 10X-Salt-SW. As shown, 10X-DOW, 10X-Salt-SW, and Mg(NO_3_)_2_ solution promoted the production of cordycepin (*p* < 0.05). However, compared with the production of cordycepin in the UPW group, the NaCl solution reduced the production of cordycepin by 57 % and had a suppressive effect (*p* < 0.001). The effect of KCl, CaCO_3_, and (NH_4_)_2_Fe(SO_4_)_2_ solutions were not significant (*p* > 0.05). Moreover, the effects of DOW, Salt-SW, and various salts solution on the production of adenosine are presented in Fig. 2b. 10X-DOW and 10X-Salt-SW enhanced the production of adenosine (*p* < 0.05). Among the salts solution, only (NH_4_)_2_Fe(SO_4_)_2_ solutions promoted the production of adenosine (*p* < 0.05).

### Effect of various sodium salts on production of cordycepin and adenosine of *Cordyceps militaris*-fermented products

**Fig. 3:**
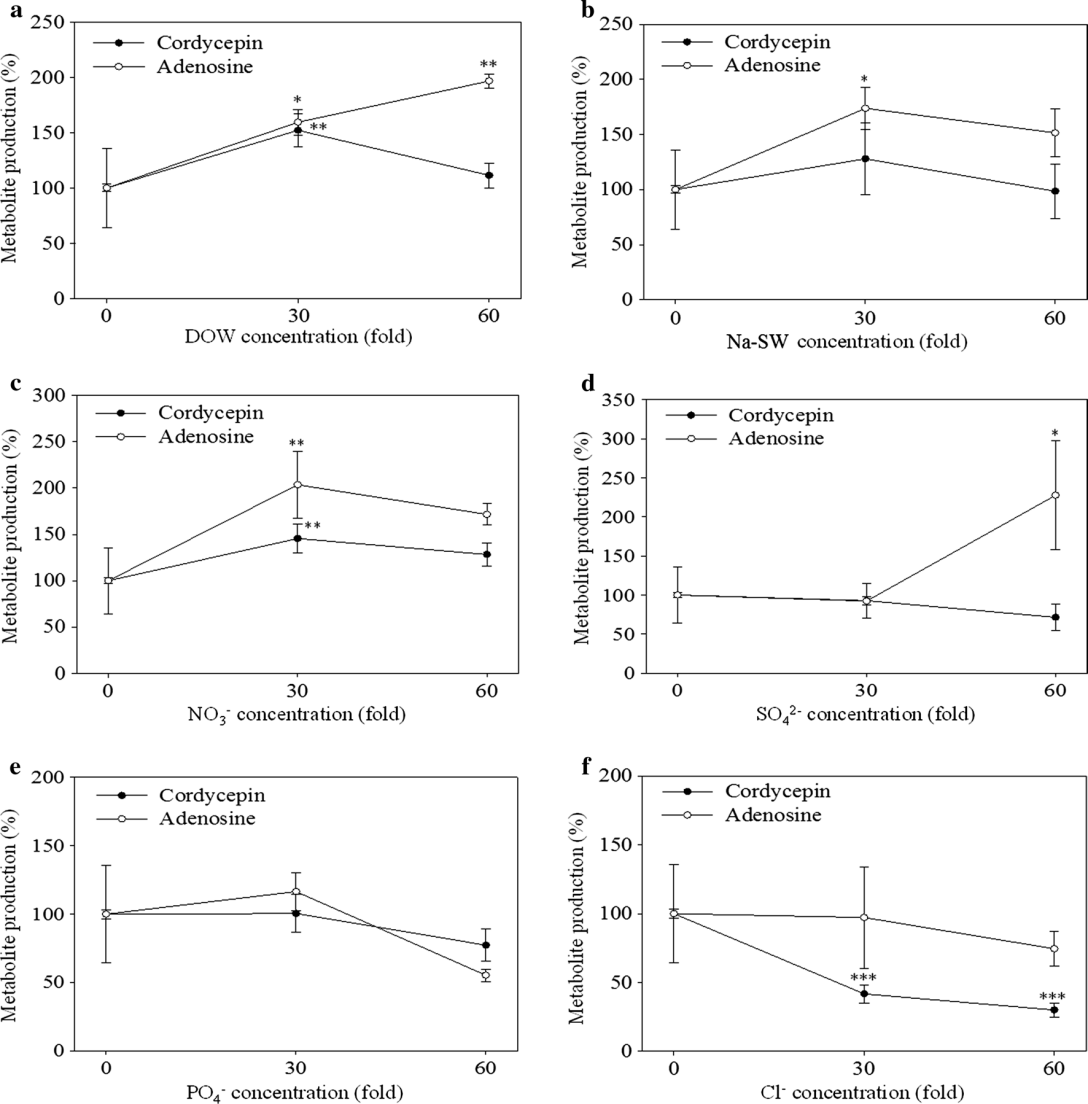
Effect of various sodium salt ions on the cordycepin and adenosine production of *C. militaris*-fermented oats in solid culture. DOW (**a**), Na-SW (**b**), NaNO_3_ (**c**), Na_2_SO_4_ (**d**), Na_3_PO_4_ (**e**), or NaCl (**f**) solution was added to the solid substrate of *C. militaris* during solid culture, respectively. NaNO_3_, Na_2_SO_4_, Na_3_PO_4_ and NaCl solution included equal SO_4_^2−^, PO_4_^3−^, NO_3_^−^, and Cl^−^ concentration to that in 30X or 60X DOW, respectively. Na-SW was a mixture of the four sodium salt solutions. Data are presented as mean ± SD (n = 3). *, **, *** indicated the significant difference (*p* < 0.05, *p* < 0.01, *p* < 0.001, respectively) as compared with UPW.

According to above results (Fig. 2a), NaCl solution significantly suppressed the production of cordycepin. This section further discusses the effect of various sodium salts on production of cordycepin and adenosine. According to the comparison in Fig. 3, at the 30-fold sodium salt concentration, DOW, Na-SW, NaNO_3_, Na_2_SO_4_, Na_3_PO_4_, and NaCl, the *C. militaris*-fermented products with the DOW source and NaNO_3_ respectively had 52 and 46 % higher cordycepin production than the *C. militaris*-fermented products with UPW (*p* < 0.01). However, after fermentation, NaCl increased cordycepin production by 58 % (*p* < 0.001). Moreover, DOW, Na-SW, and NaNO_3_ enhanced adenosine production by 60, 74, and 104 % (*p* < 0.05), respectively. Nevertheless, the selection of DOW, Na_2_SO_4_, Na_3_PO_4_, and NaCl did not affect the production of adenosine. At a concentration of 60-fold, the DOW, Na-SW, NaNO_3_, Na_2_SO_4_, and Na_3_PO_4_ groups exhibited a decline in cordycepin production. However, in contrast to the UPW group, the NaCl group showed a significant 70 % decrease in the production of cordycepin (*p* < 0.001). Furthermore, DOW and Na_2_SO_4_ increased adenosine production (*p* < 0.05), whereas adenosine production decreased in the other groups.

### Effect of various nitrate salts on production of cordycepin and adenosine in *Cordyceps militaris*-fermented products

**Fig. 4:**
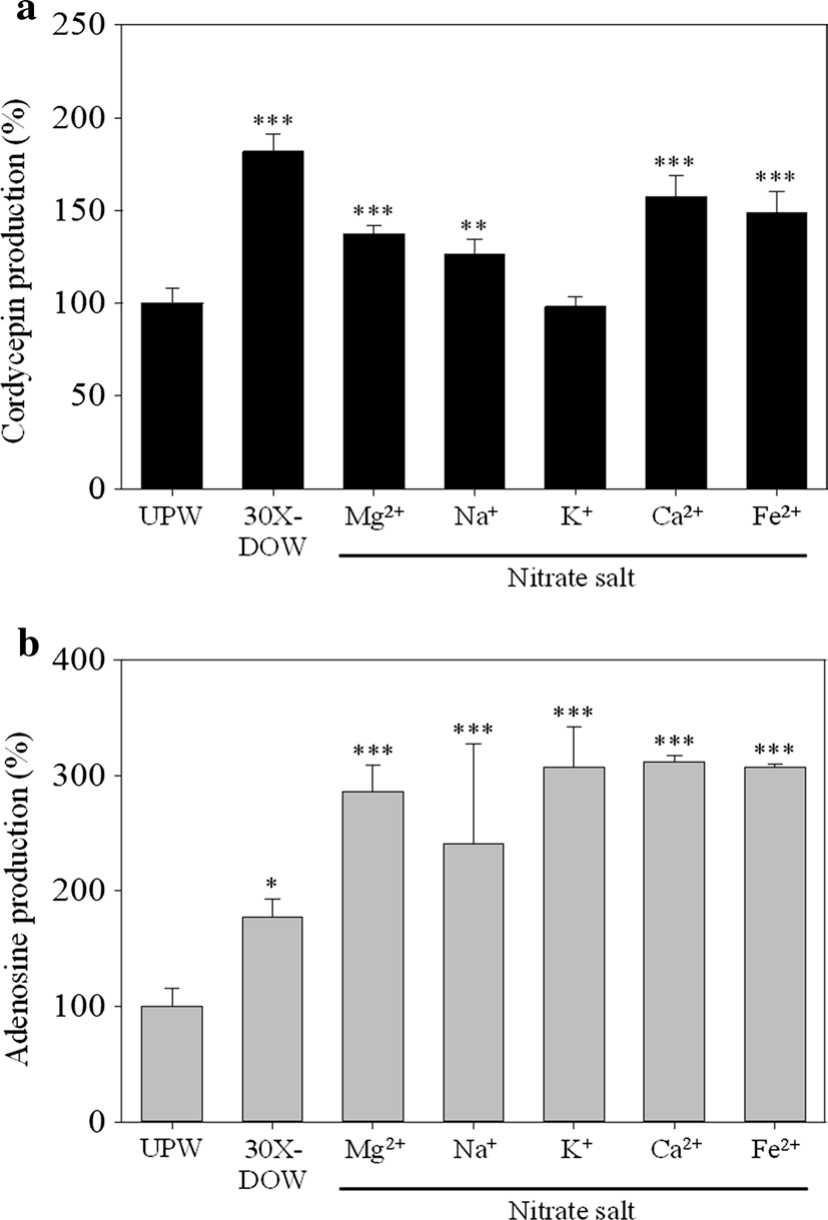
Effect of various nitrate salts on the cordycepin (**a**) and adenosine (**b**) production of *C. militaris*-fermented oats in solid culture. The 30X concentration of Mg^2+^, Na^+^, K^+^, Ca^2+^, Fe^2+^ in Mg(NO_3_)_2_, NaNO_3_, KNO_3_, Ca(NO_3_)_2_, Fe(NO_3_)_2_·6H_2_O were equal to that in 30X-DOW. Data are presented as mean ± SD (n = 3). *, **, *** indicated the significant difference (*p* < 0.05, *p* < 0.01, *p* < 0.001, respectively) as compared with UPW.

As shown in Fig. 3, at the 30-fold sodium salt concentration, only NaNO_3_ could enhance cordycepin and adenosine production. Therefore, this section focuses on the concentration of NaNO_3_ and discusses the effect of various combinations of nitrate salts on the production of cordycepin and adenosine. The 30X concentration of Mg^2+^, Na^+^, K^+^, Ca^2+^, Fe^2+^ in Mg(NO_3_)_2_, NaNO_3_, KNO_3_, Ca(NO_3_)_2_, Fe(NO_3_)_2_·6H_2_O were equal to that in 30X-DOW. According to Fig. 4a, DOW, Mg(NO_3_)_2_, NaNO_3_, Ca(NO_3_)_2_, and Fe(NO_3_)_2_ had significantly increased cordycepin production (*p* < 0.01). However, KNO_3_ did not exhibit such an increase (*p* < 0.05). Moreover, Fig. 4b illustrates that Mg(NO_3_)_2_, NaNO_3_, KNO_3_, Ca(NO_3_)_2_, and Fe(NO_3_)_2_ significantly increased adenosine production (*p* < 0.001), whereas 30X-DOW moderately increased adenosine production (*p* < 0.05).

### Effect of various chloride salts on production of cordycepin and adenosine in *Cordyceps militaris*-fermented products

**Fig. 5:**
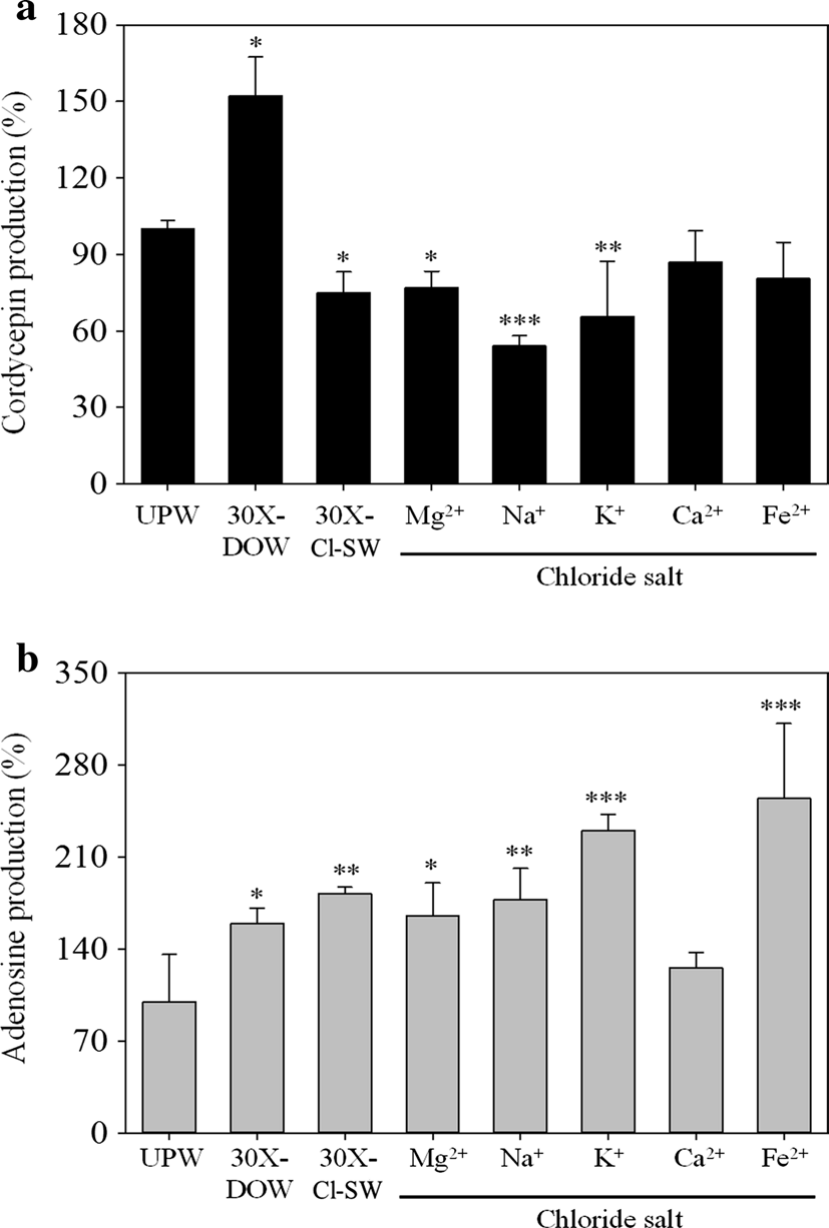
Effect of various chloride salts on the cordycepin (**a**) and adenosine (**b**) production of *C. militaris*-fermented product in solid culture. The 30X concentration of Mg^2+^, Na^+^, K^+^, Ca^2+^, Fe^2+^ in MgCl_2_, NaCl, KCl, CaCl_2_, FeCl_2_ were equal to that in 30X-DOW. Cl-SW was a mixture of the five chloride salt solutions. Data are presented as mean ± SD (n = 3). *, **, *** indicated the significant difference (*p* < 0.05, *p* < 0.01, *p* < 0.001, respectively) as compared with UPW.

Figure 4 illustrates that at the 30-fold sodium salt concentration, NaNO_3_ enhanced cordycepin production, but Na_2_SO_4_ and NaPO_4_ did not enhance cordycepin production. However, NaCl significantly reduced cordycepin production (*p* < 0.001). Therefore, this section uses solutions with equal metal ion concentrations to that in 30X-DOW to discuss the effect of various chloride salts on production of cordycepin and adenosine. As shown in Fig. 5a, MgCl_2_, NaCl, and KCl significantly reduced cordycepin production (*p* < 0.05), and cordycepin production in the CaCl_2_ and FeCl_2_ solutions declined. The Cl-SW solution produced by combining all five chloride salts significantly reduced cordycepin production (*p* < 0.05).

The adenosine production results are shown in Fig. 5b. DOW and Cl-SW enhanced the production of adenosine (*p* < 0.05). Furthermore, MgCl_2_, NaCl, KCl, and FeCl_2_ significantly enhanced the production of adenosine (*p* < 0.05), whereas CaCl_2_ had no significant effect on adenosine production (*p* < 0.05). However, Cl-SW enhanced adenosine production (*p* < 0.05).

## Discussion

As confirmed in previous studies, DOW can enhance production of the functional ingredients of red mold dioscorea such as monascin and ankaflavin as well as reduce the production of hepatotoxin, nephrotoxin, and citrinin (Lee et al. 2011). DOW can also promote production of *A. camphorata* mycelium as well as increase production of the functional ingredients of triterpenoids, polysaccharide, and flavonoids (Wang et al. 2013a). This study focuses on submerged culture to investigate the effect of DOW on production of *C. militaris* mycelium and the functional ingredients of *C. militaris*. Table 1 demonstrates that DOW promoted the production of *C. militaris* mycelium more than UPW did and significantly increased the production of adenosine (*p* < 0.05). This result agrees with those of previous studies except for DOW significantly affecting the production of intracellular and extracellular β-1,3-glucan (*p* < 0.05), a phenomenon that was not observed in this study.

Moreover, this study addressed the effect of DOW of various concentrations on the production of cordycepin and adenosine in *C. militaris*-fermented products. In this study, cordycepin production increased as the concentration of DOW increased. The content of cordycepin in the 30X-DOW solution reached the highest point on the 80th day of fermentation (*p* < 0.01). The DOW of various concentrations did not significantly enhance the production of adenosine (*p* > 0.05). The results were compared with the SW results. Mg(NO_3_)_2_ promoted cordycepin production (*p* < 0.05), NaCl significantly suppressed cordycepin production (*p* < 0.001), and KCl reduced cordycepin production. However, NaCl and KCl had Cl^−^, whereas CaCO_3_ and (NH_4_)_2_Fe(SO_4_)_2_ had no significant effect on cordycepin production (*p* > 0.05). Furthermore, SW can increase cordycepin production to a degree similar to that of DOW. (NH_4_)_2_Fe(SO_4_)_2_ increases adenosine production (*p* < 0.05). However, the other four salt solution did not have this effect (*p* > 0.05). SW can also increase adenosine production to a degree similar to that of DOW.

The results illustrated that all the metal ions in DOW can either increase or not affect cordycepin production. However, the production of cordycepin in NaCl solution significantly decreases after fermentation. Na^+^ constitutes a large proportion of DOW. This study compared the effects of DOW and various sodium salt solutions with concentrations equal to that of DOW on the production of cordycepin and adenosine. At the 30-fold sodium salt concentration, NO_3_^−^ increased the production of cordycepin (*p* < 0.01) and adenosine (*p* < 0.05) the most; however, it had no effect on adenosine production. SO_4_^2−^ and PO_4_^−^ had no effect on the production of cordycepin and adenosine. SW did not significantly increase cordycepin production but significantly increased the production of adenosine (*p* < 0.05). At the 60-fold sodium salt concentration, the production of cordycepin and adenosine with SW, NO_3_^−^, and PO_4_^−^ decreased. At this concentration, the cordycepin production with SO_4_^2−^ declined as well, but the adenosine production increased significantly (*p* < 0.05). Concurrently, Cl^-^ of the 60-fold concentration significantly reduced cordycepin production (*p* < 0.001). The NO_3_^−^, SO_4_^2−^, PO_4_^−^, and Cl^−^ used in this study refer to sodium salts. The NaCl solution reduced cordycepin production, whereas the NaNO_3_ solution increased cordycepin production. Therefore, we conclude that NO_3_^−^ can increase cordycepin production, that Cl^−^ may reduce cordycepin production, and that NaCl solution has no effect on adenosine production. Nevertheless, the NaNO_3_ solution promoted adenosine production. At the 60-fold sodium salt concentration, only the Na_2_SO_4_ solution enhanced adenosine production. Therefore, NO_3_^−^ and SO_4_^2−^ may enhance adenosine production, whereas Cl^−^ does not affect adenosine production.

Regarding to the effect of nitrates of various compositions on the production of cordycepin and adenosine (Fig. 4), Mg(NO_3_)_2_, NaNO_3_, Ca(NO_3_)_2_, and Fe(NO_3_)_2_ significantly increased cordycepin production (*p* < 0.01). However, NaNO_3_ did not have this effect (*p* > 0.05). Mg(NO_3_)_2_, NaNO_3_, KCl, Ca(NO_3_)_2_, and Fe(NO_3_)_2_ significantly increases adenosine production (*p* < 0.001). Because equal amounts of NO_3_^−^ produces equal effects, Mg^2+^, Na^+^, Ca^2+^, and Fe^2+^ are estimated to be primarily responsible for increasing production of cordycepin and adenosine. In addition, K^+^ is likely to significantly increase adenosine production.

According to Fig. 5, Cl^−^ can suppress cordycepin production. This study used various chloride salts to investigate the effect of metal ions on the production of cordycepin and adenosine. All the chloride salts, MgCl_2_, NaCl, and KCl significantly reduced cordycepin production (*p* < 0.05). Furthermore, CaCl_2_ and FeCl_2_ reduced cordycepin production. Therefore, the SW composed of the five chloride salts significantly reduced cordycepin production (*p* < 0.05). Furthermore, MgCl_2_, NaCl, KCl, and FeCl_2_ significantly increased adenosine production (*p* < 0.05). However, CaCl_2_ did not increase adenosine production (*p* > 0.05). SW increased adenosine production (*p* < 0.05). The addition of Mg(NO_3_)_2_, NaNO_3_, KNO_3_, Ca(NO_3_)_2_, or Fe(NO_3_)_2_ can significantly enhance cordycepin production. However, in all the chloride salts, MgCl_2_, NaCl, KCl, CaCl_2_, and FeCl_2_ either significantly reduced cordycepin production or caused a declining trend in cordycepin production. Therefore, Cl^−^ might contribute to reducing cordycepin production in *C. militaris*-fermented products.

The effect of SW on the production of cordycepin and adenosine varied depending on the combination of sodium salts. As shown in Fig. 3, Na-SW composed of various sodium salts had no significant effect on cordycepin production but enhanced adenosine production. The increase of cordycepin production might have been attributed to NaNO_3_. Moreover, NaCl suppressed the production of cordycepin, and the effect of Na_2_SO_4_ and NaPO_4_ on cordycepin production was not significant. Therefore, the combined effect of the four sodium salts rendered the resultant Na-SW least effective for promoting cordycepin production. Moreover, NaNO_3_ enhanced adenosine production, whereas Na_2_SO_4_, NaPO_4_, and NaCl had no effect on adenosine production. Hence, the combination of these four sodium salts resulted in the Na-SW that significantly promoted adenosine production. Figure 5a, b show that the Cl-SW of various combinations of chloride salts suppressed cordycepin production and promoted adenosine production. This Cl-SW effect might have occurred because MgCl_2_, NaCl, and KCl were all active in suppressing cordycepin production, and CaCl_2_ and FeCl_2_ were not significantly associated with cordycepin production. Therefore, the Cl-SW comprising the five chloride salts reduced cordycepin production. Moreover, except for CaCl_2_, the other chloride salts increased adenosine production. Therefore, the Cl-SW increased adenosine production. As confirmed in Figs. 2 and 5 demonstrate that Salt-SW increased the production of cordycepin and adenosine, and the Mg(NO_3_)_2_ sodium salt should be the functional salt for increasing the production of cordycepin and adenosine. The results of fermentation in various chloride salt mediums revealed that NaCl and KCl suppressed cordycepin production but enhanced adenosine production. This result is verified in Fig. 5a, b. However, CaCO_3_ and (NH_4_)_2_Fe(SO_4_)_2_ least enhanced cordycepin production, whereas (NH_4_)_2_Fe(SO_4_)_2_ increased adenosine production (Fig. 2). Figure 4a, b imply that Ca^2+^ and Fe^2+^ are likely to enhance the production of cordycepin and adenosine. Hence, the Salt-SW comprising Mg(NO_3_)_2_, NaCl, KCl, CaCO_3_, and (NH_4_)_2_Fe(SO_4_)_2_ enhanced production of cordycepin and adenosine. Furthermore, Salt-SW produced by combining sodium and chloride salts increased adenosine production to the same extent that DOW did. However, the Na-SW comprising only sodium salts increased cordycepin production to nearly the same extent that DOW did.

The study by Cui and Zhang (2012) noted that the addition of Mg^2+^ and Mn^2+^ in the cultivation of *C. militaris* in submerged culture significantly promoted *C. militaris* mycelium and extracellular polysaccharide production. Moreover, adding sodium selenite into the solid culture mediums for *C. militaris* cultivation significantly increased the production of cordycepin, cordyceps acid, cordyceps polysaccharide, and organic selenium of the *C. militaris* fruit body compared with the control group that had no sodium selenite. This production increase was proportional to the concentration of sodium selenite (Dong et al. 2012). The addition of 10 mM Ca^2+^ in *Ganoderma* liquid culture mediums can significantly increase the production of Ganoderma acid (Xu and Zhong 2012). In summary, compared with UPW, DOW can more effectively promote the growth of *C. militaris* mycelium and enhance the production of cordycepin, its functional ingredient. Moreover, the concentration of DOW has a dose effect on cordycepin production. The enhancing effect of DOW on cordycepin production is mainly attributed to its ions such as Mg^2+^, Na^+^, Ca^2+^, Fe^2+^, and NO_3_^−^. However, the Cl^−^ in DOW suppresses cordycepin production. For adenosine, Mg^2+^, Na^+^, K^+^, Ca^2+^, Fe^2+^ and SO_4_^2−^ have the ability of increasing its production, but such an ability is not observed for Cl^−^. Furthermore, the DOW ions can increase cordycepin production. This ability might be induced by the pressure of the *C. militaris* growth environment, which forces *C*. *militaris* to enter the secondary metabolic phase and begin producing secondary metabolites. This ability could stem from ions in DOW that serve as enzyme or protein cofactors in cultivating *C. militaris* and produce cordycepin to promote the growth of mycelium and production of the functional ingredients of *C. militaris*. The effect of Na-SW on the production of cordycepin and adenosine varies depending on the combination of sodium salts. Therefore, the production of cordycepin can be increased by adding nitrates and reducing the production of Cl^−^ in DOW. Moreover, chloride salts, nitrates, and SO_4_^2−^ can be added to DOW to increase adenosine production.

This study employed DOW to cultivate *C. militaris* in submerged and solid culture and investigated the effect of DOW on the production of *C. militaris*’ fermentative products, namely cordycepin and adenosine. The results demonstrated that using 30X-DOW as a water source to produce *C. militaris*-fermented products can significantly increase the production of cordycepin. This cordycepin production is 65 % higher than the *C. militaris*-fermented products of the UPW water solution (*p* < 0.001). The concentration of DOW also has a dose effect on cordycepin production. Moreover, the Mg^2+^, Na^+^, Ca^2+^, Fe^2+^, and NO_3_^−^ in DOW increase cordycepin production; however, the Cl^−^ in DOW exhibits an opposite effect. Mg^2+^, Na^+^, K^+^, Ca^2+^, Fe^2+^, and SO_4_^2−^ can increase adenosine production, but Cl^−^ cannot. The Cl-SW containing MgCl_2_, NaCl, KCl, CaCl_2_, FeCl_2_ can not only increase adenosine production but also promote cordycepin production to nearly the same extent that DOW does.
